# Maternal diet in pregnancy and the risk of inflammatory bowel disease in the offspring: a prospective cohort study

**DOI:** 10.1016/j.ajcnut.2024.10.017

**Published:** 2024-10-24

**Authors:** Annie Guo, Anne Lise Brantsæter, Tiril Cecilie Borge, Elin M Hård af Segerstad, Henrik Imberg, Karl Mårild, Ketil Størdal

**Affiliations:** 1 Department of Pediatrics, Institute of Clinical Sciences, Sahlgrenska Academy, University of Gothenburg, Gothenburg, Sweden; 2 Department of Food Safety and Centre for Sustainable Diets, Norwegian Institute of Public Health, Oslo, Norway; 3 Cluster for Reviews and Health Technology Assessments, Norwegian Institute of Public Health, Oslo, Norway; 4 Pediatric Research Institute, Oslo University Hospital and Faculty of Medicine, University of Oslo, Oslo, Norway; 5 Statistiska Konsultgruppen Sweden, Gothenburg, Sweden; 6 Department of Molecular and Clinical Medicine, Institute of Medicine, Sahlgrenska Academy, University of Gothenburg, Gothenburg, Sweden; 7 Department of Pediatrics, Queen Silvia Children’s Hospital, Gothenburg, Sweden; 8 Department of Child and Adolescent Health, Oslo University Hospital, Oslo, Norway

**Keywords:** ulcerative colitis, Crohn disease, MoBa, pregnancy

## Abstract

**Background:**

Diet has been hypothesized as a risk factor for the development of inflammatory bowel disease (IBD).

**Objective:**

The objective of this study was to explore associations between maternal diet diversity and quality in pregnancy and the offspring’s risk of IBD.

**Methods:**

We used data from a nationwide cohort study on 85,129 Norwegian children followed from birth (1999–2009) with information on maternal diet in pregnancy from validated food frequency questionnaires. Hazard ratios (HRs) for IBD, Crohn disease (CD), and ulcerative colitis (UC) by maternal diet diversity, quality, and intake amounts of individual food groups were adjusted for maternal BMI, parental IBD, and sociodemographic factors. Sensitivity analyses were adjusted for the child’s early-life diet quality and antibiotic treatment. Dietary exposures were classified into tertiles, comparing low (reference) with medium, and high levels.

**Results:**

During a mean follow-up time of 16.1 y (1,367,837 person-years of follow-up), 268 children developed IBD (CD, *n* = 119; UC, *n* = 76; IBD-unclassified, *n* = 73). High compared with low diet diversity in pregnancy was associated with a lower risk of UC in the offspring [adjusted HR (aHR) 0.46, 95% confidence interval: 0.25, 0.87], with consistent findings after further adjustment for the child’s early-life diet quality and antibiotic treatment. High compared with low diet diversity in pregnancy yielded aHRs of 0.81 for CD (0.51–1.28) and 0.75 for any IBD (0.55–1.02) in the offspring. A high compared with low diet quality in pregnancy or intakes of specific food groups were not associated with the offspring’s risk of IBD or its subtypes.

**Conclusions:**

Our findings suggest that a higher maternal diet diversity in pregnancy may be associated with a lower risk of UC in the offspring.

## Introduction

The prevalence of inflammatory bowel disease (IBD), which includes Crohn’s disease (CD) and ulcerative colitis (UC), has shown a significant rise during the last decades [1]. It is believed that the development and increased occurrence of IBD may be attributed to alterations in environmental exposures, the gut microbiome, and a dysregulated immune response in susceptible individuals [2].

Previous research has indicated that diet may play a role in the development of IBD. Although adherence to dietary patterns [3,4] and intakes of specific food groups [[5], [6], [7]] have been associated with the risk of IBD, a varied diet is also advocated by international guidelines [8]. Diet diversity is traditionally described as consuming a variety of foods and is suggested to favor the diversity of the gut microbiome [9], which may be of particular importance for IBD. Although early-life diet in the first year of life has been suggested to influence IBD risk [10], it is hypothesized that diet in the gestational period also affects the individual’s risk of disease later in life [11,12]. Maternal diet in pregnancy has been shown to influence the neonatal microbiome as well as the immune system, and has been linked to long-term consequences for the offspring’s health [13,14], including the risk of immune-mediated diseases such as celiac disease [15], atopy [16], and type 1 diabetes [17].

Although ongoing research aims to assess dietary intervention during pregnancy and offspring’s risk of IBD [12], prospectively collected data on diet in pregnancy and investigation of the child’s risk of IBD are scarce. In a nationwide pregnancy cohort study, we aimed to explore associations of maternal diet diversity, quality, and intakes of specific food groups in pregnancy with the offspring’s risk of developing IBD and its subtypes.

## Methods

### Study population

**FIGURE 1:**
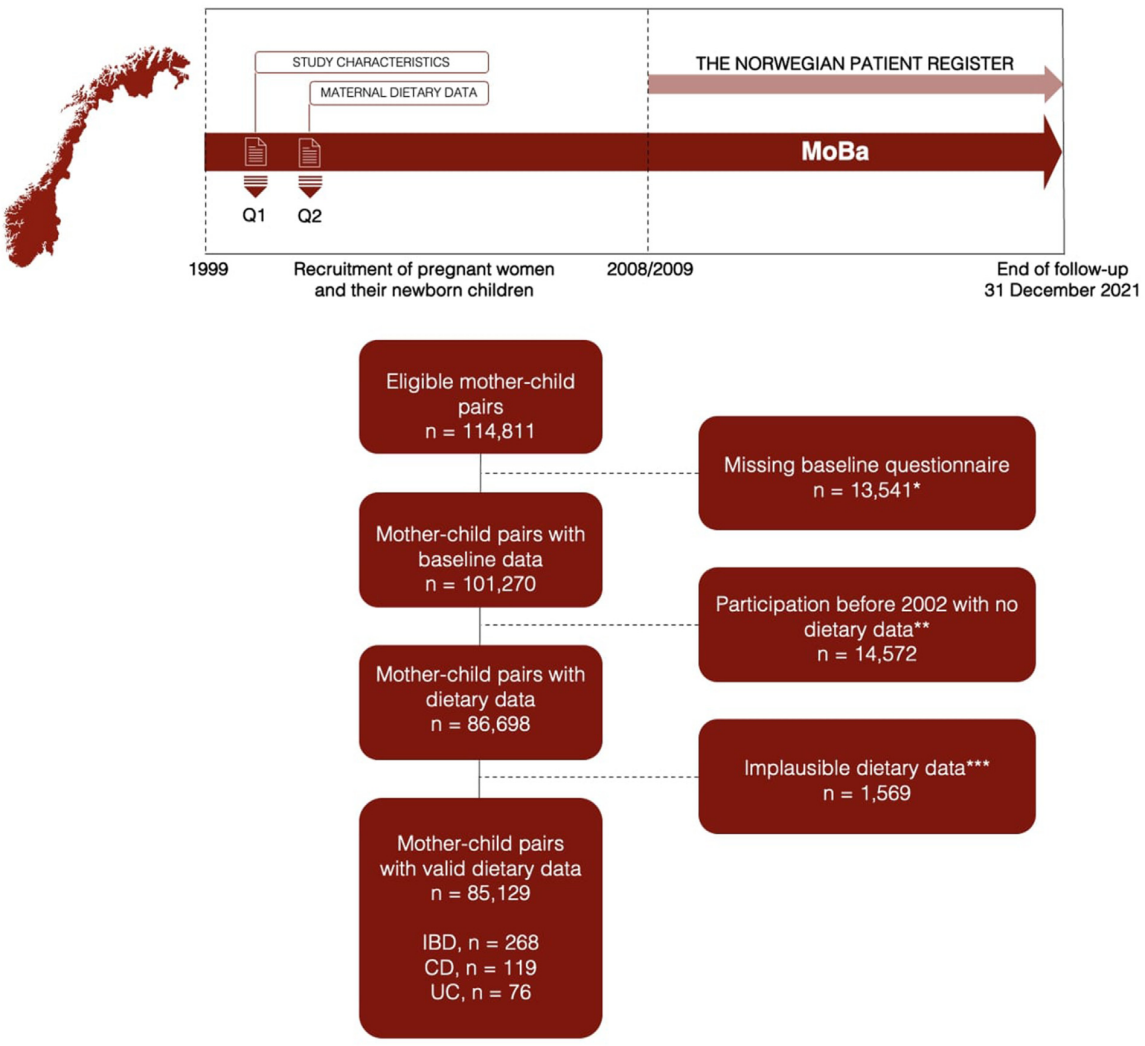
Flowchart of the included participants in the Norwegian Mother, Father, and Child Cohort Study (MoBa). ∗Children not registered in the Medical Birth Registry of Norway with a live birth, died <12 mo of age or emigrated ∗∗The MoBa food frequency questionnaire (FFQ) was first introduced in 2002, ∗∗∗Defined as energy intake <4.5 megajoules (MJ) or >20 MJ, and >3 blank pages in the MoBa FFQ [19]. The definition of inflammatory bowel disease (IBD) included Crohn disease (CD), ulcerative colitis (UC), and IBD-unclassified (Supplemental Table 4).

We retrieved data from the Norwegian Mother, Father, and Child Cohort Study (MoBa), a pregnancy cohort comprising ∼114,500 children, 95,200 mothers, and 75,200 fathers. Participants were recruited from all over Norway; of those invited, 41% consented to participate (Supplemental File, page 2) [18]. Inclusion in this study was restricted to children born to mothers who had completed the baseline questionnaire collected in gestational week (GW) 15 and a food frequency questionnaire (FFQ) administered in GW 22 during the years 2002–2008. After excluding children born to mothers with incomplete questionnaires collected in GW 15 (*n* = 13,541), who were recruited before the inclusion of the FFQ or had not answered the FFQ (*n* = 14,572), or who reported implausible dietary data (*n* = 1569), 85,129 children remained in the study (Figure 1) [19]. Individual-level data were retrieved from the Norwegian Patient Registry [20] and the Medical Birth Registry of Norway [21].

### Maternal diet in pregnancy

#### FFQ

A semiquantitative FFQ was developed and validated for pregnant females in MoBa and used from 2002 throughout the recruitment period. Participants were asked to report their average intake of 255 food and beverage items since becoming pregnant [19]. The FFQ was validated in a subsample of 119 MoBa participants and showed fair agreement with a 4-d weighed food dairy and biomarkers in urine and blood samples [[22], [23], [24], [25]]. It has been widely used to examine associations between maternal diet during pregnancy and health outcomes in mothers and children, with the exposure modeled as maternal dietary patterns, food indices, or the estimated amount of specific foods or nutrients [[26], [27], [28]]. Food frequencies were converted into daily intakes (g/d), and FoodCalc [29] and the Norwegian food composition table (version 2005) were used to calculate nutrient and energy intakes. As in previous studies [19], we excluded individuals with implausible dietary data (energy intake <4.5 MJ or >20 MJ) and with an incomplete FFQ (>3 blank pages, Figure 1). We did not examine dietary supplements during pregnancy.

#### Maternal diet diversity and quality

To examine the influence of a diverse diet in pregnancy, we used a diet diversity index based on the Revised Diet Quality Index [30]. It is 1 of the components used in the Prenatal Diet Quality Index (PDQI) [31], previously used to study prenatal diet and offspring’s disease outcomes [31,32]. The diet diversity index captures the intake variety across 25 food items, where the mothers are being allocated a positive score if they had a regular intake of at least 1/4 of a serving of each respective food item (Supplemental Table 1). The 25 food items were then weighted into 4 food groups: grains, vegetables, fruits, and animal-based products. Within each food group, the score reflects the percentage of the possible maximum score of 2.5. For example, although a mother consuming at least 1/2 of a serving in only 3 of 8 possible grain categories would receive a score of 3/8 × 2.5 or 0.94 of 2.5 points, a mother consuming 7 of 8 possible grain categories would be allocated a score of 7/8 × 2.5 or 2.2 of 2.5 points. Finally, the 4 subgroups were summarized into a continuous diet diversity index with a total maximum score of 10. This study analyzed the diet diversity component as a separate exposure variable divided by tertiles.

To define maternal diet quality, we used a modified version of the PDQI that assesses the adherence to the Norwegian dietary guidelines [31]. This index is based on the healthy eating index (HEI) as outlined in the United States dietary guidelines [33] and aligns with other international nutritional guidelines [34]. To adjust the index to the scope of this study, we modified the PDQI by omitting the “meal pattern” component. Thus, the index used in this study comprised 12 components, including diet diversity, as described in Supplemental Table 2. The total index was determined by summing the scores of all components, with higher values indicating higher adherence to the dietary guidelines (i.e., higher intakes of fruits, vegetables, whole grains, and fish, a more diverse diet, and lower intakes of saturated fat, salt, and added sugar). The modified PDQI had a maximum value of 110 and we divided it by tertiles to classify participants into low, medium, and high levels.

#### Maternal intake of food groups

Motivated by previous research assessing food intakes and the risk of IBD [5,6,35], we predefined maternal intake of 11 food groups: red meat, white meat, fatty fish, lean fish and seafood, dairy, vegetables, whole grains, refined grains, salty foods, sugary foods, and drinks (Supplemental Table 3). We divided the amount of each food group by tertiles, indicating low, medium, and high intake.

#### IBD

The outcomes of this study were IBD, CD, and UC, which we defined as having at least 2 International Statistical Classification of Diseases and Health Related Problems Tenth Revision codes for IBD and the subtypes in the Norwegian Patient Registry [20]. Diagnostic data were collected until 31 December, 2021 (Supplemental Table 4). According to a review of medical records, this IBD definition has demonstrated a positive predictive value of ≥93% for IBD in Norway [36]. Although we defined subtype-specific IBD codes for CD and UC, a mix of IBD codes was included in the analyses of any IBD and classified as IBD-unclassified (IBD-U).

### Other data

We used Norwegian registries [20,21] and the baseline questionnaire administered at GW 15 to retrieve data on the following covariates: child’s sex, parental IBD, maternal origin, maternal education level, maternal immune-mediated comorbidities, and prepregnancy BMI (Supplemental Table 5). Additional information on the child’s diet quality (low, medium, or high) at 18 mo of age was assessed using an HEI modified for children [10]. Here, high diet quality represented higher intakes of fruits and vegetables, fish, and dairy foods and lower intakes of meat, soft drinks, and salty and sweet snacks. We also collected data on maternal antibiotic use in pregnancy (yes/no) and the child’s antibiotic use by 18 mo of age (yes/no). A directed acyclic graph of potential confounders, mediators, and ancestors of outcome is provided in Supplemental Figure 1. Covariates were categorized as shown in Table 1.

**TABLE 1:** Characteristics of the study participants (*n* = 85,129).

	Maternal diet diversity in pregnancy		
	Low (*n* = 28,623)	Medium (*n* = 29,603)	High (*n* = 26,903)
Child characteristics			
Sex			
Female	13,297 (48.7)	14,477 (48.9)	13,175 (49.0)
Male	14,696 (51.4)	15,126 (51.1)	13,728 (51.0)
Diagnosis			
IBD^1^	110 (0.4)	88 (0.3)	70 (0.3)
CD	47 (0.2)	41 (0.1)	31 (0.1)
UC	36 (0.1)	24 (0.1)	16 (0.1)
Child’s diet quality at 18 mo^2^			
Low	4963 (27.4)	4064 (22.0)	3116 (18.3)
Medium	9599 (53.0)	9842 (53.2)	9043 (53.1)
High	3545 (19.6)	4597 (24.8)	4871 (28.6)
Parental characteristics			
Maternal diet quality^3^			
Low	17,475 (61.1)	8578 (29.0)	2321 (8.6)
Medium	7906 (27.6)	11,920 (40.3)	8547 (31.8)
High	3242 (11.3)	9105 (30.8)	16,035 (59.6)
Parental IBD	689 (2.4)	714 (2.4)	628 (2.3)
Maternal origin^4^			
Norway	27,526 (96.2)	28,194 (95.2)	24,799 (92.2)
Maternal education (y)			
≤11	2674 (9.3)	1845 (6.2)	1484 (5.5)
12	9468 (33.1)	7964 (26.9)	6845 (25.4)
≥13	16,356 (57.1)	19,671 (66.4)	18,438 (68.5)
Missing data	125 (0.4)	123 (0.4)	136 (0.5)
Maternal comorbidities^5^			
Yes	1121 (3.9)	1247 (4.2)	1129 (4.2)
Maternal prepregnancy BMI			
Median (IQR)	23.4 (21.3–26.5)	23.1 (21.2–25.9)	22.9 (21.0–25.5)
Mean (SD)	24.4 (4.5)	24.0 (4.2)	23.7 (4.1)

Abbreviations: CD, Crohn’s disease; IBD, inflammatory bowel disease; IQR, inter quartile range; SD, standard deviation; UC, ulcerative colitis.

Presented as number (%) if not otherwise stated.

1 Including CD, UC, and IBD-unclassified (defined as individuals with a mix of IBD codes).

2 Children with available data on maternal diet quality in pregnancy and diet quality at 18 mo (*n* = 53,640) calculated by using a modified healthy eating index score [10].

3 Comprises 12 components, including diet diversity, as described in Supplementary Table 2. The total score ranges from 0 to 110 points, where a higher score indicates higher diet quality. We divided it by tertiles to classify participants into low, medium, and high levels.

4 Defined by mother’s native language (Norwegian/other country).

5 Including diabetes (insulin-treated diabetes before or during pregnancy), autoimmune thyroid disease and/or rheumatoid arthritis.

### Statistical analyses

We used Poisson regression to estimate the IBD, CD, and UC incidence rates and Cox regression to estimate hazard ratios (HRs) of IBD, CD, and UC with 95% confidence intervals (CIs). The follow-up started at the child’s birth and concluded at the first IBD diagnosis or censoring at the end of the study (31 December 2021). Subanalyses for the outcome CD ignored events of other IBD subtypes, and vice versa in the UC-specific analyses. Because of the limited number of events and the higher risk of misclassification, we did not conduct a separate analysis of IBD-U. We tested the proportional hazards assumption using Schoenfeld residuals [37], a graphical data assessment, and explored interactions with time. The proportional hazards assumption was found valid for all analyses except for the intake of whole grains. We accounted for dependency between siblings in the study (*n* = 17,502) using robust SEs [38]. In our primary analyses, we designated the lowest category of diet diversity, quality, or intake of specific food groups as the reference group and compared with the higher categories. We also examined per increase in category, that is*,* from low to medium and medium to high (dose–response).

We adjusted all analyses for child and maternal characteristics previously described. Additionally, we performed sensitivity analyses in which we also adjusted for the following: *1*) the child’s diet quality at 18 mo of age, *2*) maternal antibiotic use in pregnancy, and *3*) the child’s antibiotic use by 18 mo of age. Moreover, we re-ran our main analyses for childhood-onset IBD, excluding 5 participants diagnosed aged at ≥18 y. Statistical analyses were performed using the R language and environment for statistical computing (R Core Team) version 4.1.3, including the R-packages survival (3.4-0) and survminer (0.4.9).

## Ethical statement

Informed consent was obtained by all participants. Participants in MoBa include mothers with informed written consent or mothers who had responded to questionnaires or donated blood or urine. The Regional Committees for Medical and Health Research Ethics approved the substudy in 2020 (REK 153328). The establishment of MoBa and the initial data collection were based on a license from the Norwegian Data Protection Agency and approval from the Regional Committees for Medical and Health Research Ethics. The Norwegian Health Registry Act currently regulates the MoBa cohort.

## Results

Table 1 shows the characteristics of the 85,129 children (48.8% females) followed for 1,367,837 person-years (PYR) in this study. In total, 268 children developed IBD, yielding an incidence rate of 19.6 events per 100,000 PYR (Supplemental Table 6). The mean follow-up time was 16.1 y [standard deviation (SD): 1.9 y] and the mean age at diagnosis of IBD was 12.4 y (SD: 3.7). The mean PDQI score was 81.2 (SD: 9.6, median 81.7, range 37.5–106.0). Compared with children in the lowest level of maternal diet diversity, children in the highest third more often had mothers with higher diet quality (11.3% compared with 59.6%) and ≥13 y of education (57.1% compared with 68.5%). Other characteristics, including parental IBD, maternal origin, and prepregnancy BMI, were largely similar across the groups of maternal diet diversity (Table 1).

### Maternal diet diversity and quality

**FIGURE 2:**
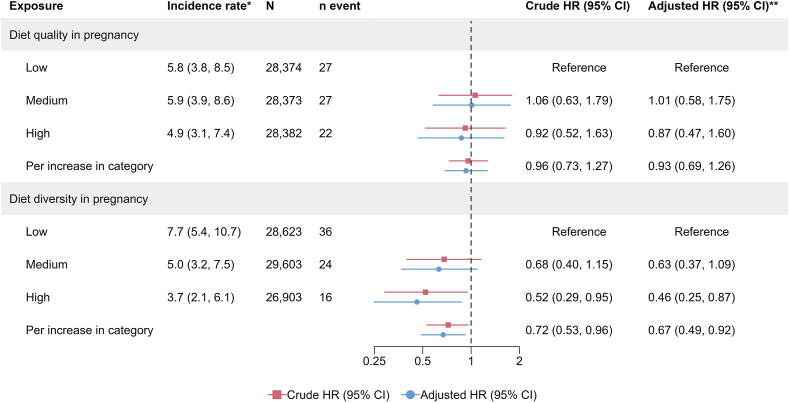
Associations between maternal diet diversity and quality in pregnancy and the risk of ulcerative colitis in the offspring. ∗Incidence rate per 100,000 person-years. **∗∗**Adjusted for the child’s sex, parental IBD, and the mother’s origin, education level, comorbidities, and prepregnancy BMI (Supplemental Table 5). CI, confidence interval; HR, hazard ratio; IBD, inflammatory bowel disease.

**FIGURE 3:**
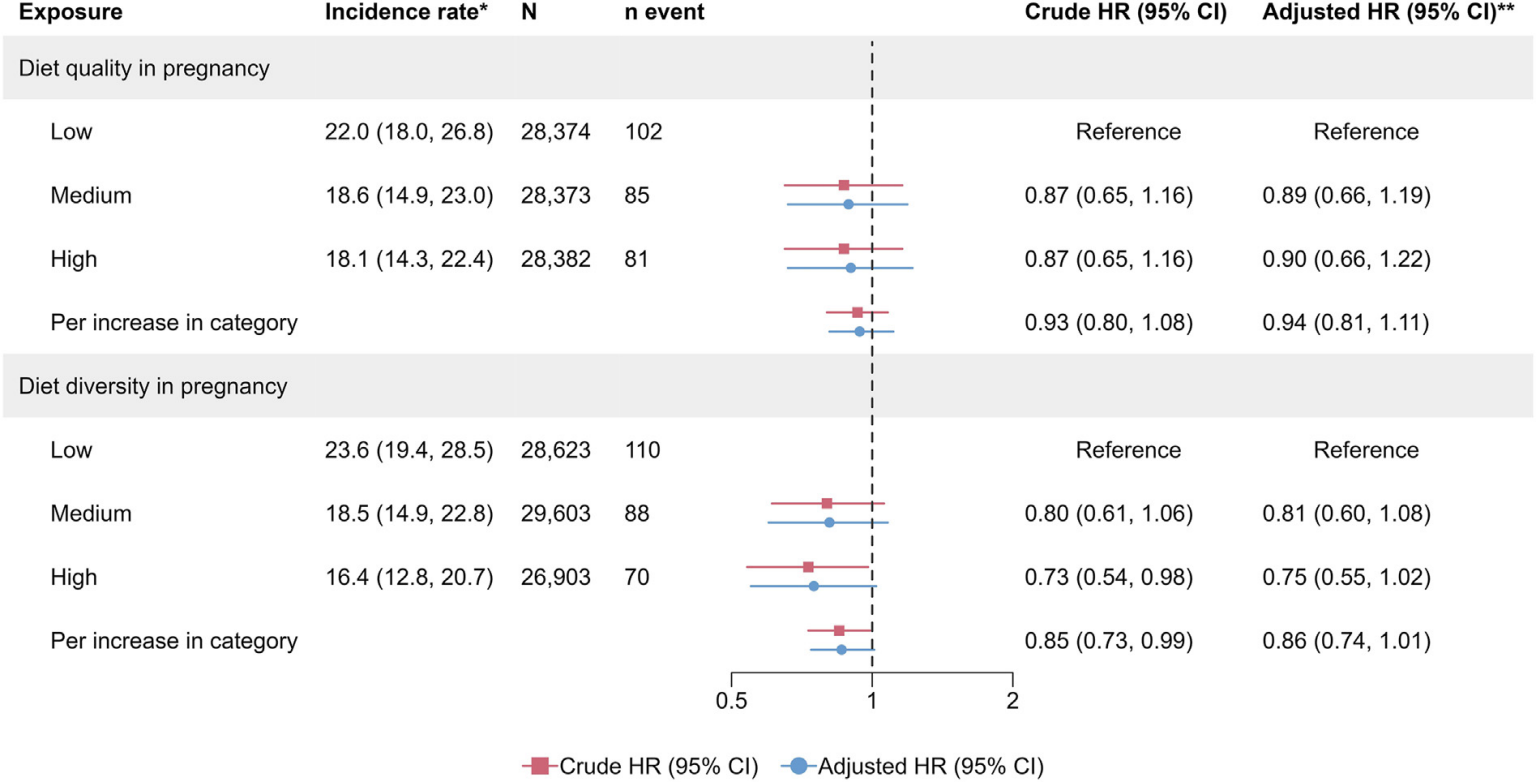
Associations between maternal diet diversity and quality in pregnancy and the risk of inflammatory bowel disease in the offspring. ∗Incidence rate per 100,000 person-years. **∗∗**Adjusted for the child’s sex, parental IBD, and the mother’s origin, education level, comorbidities, and prepregnancy BMI (Supplementary Table 5). CI, confidence interval; HR, hazard ratio; IBD, inflammatory bowel disease.

**FIGURE 4:**
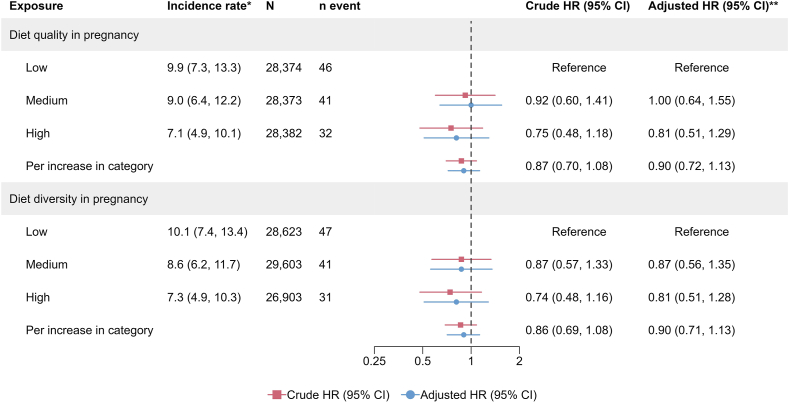
Associations between maternal diet diversity and quality in pregnancy and the risk of Crohn disease in the offspring. ∗Incidence rate per 100,000 person-years. **∗∗**Adjusted for the child’s sex, parental IBD, and the mother’s origin, education level, comorbidities, and prepregnancy body mass index (Supplemental Table 5). CI, confidence interval; HR, ratio; IBD, inflammatory bowel disease.

A high compared with low maternal diversity was associated with a lower risk of offspring UC (HR: 0.52; 95% CI: 0.29, 0.95; per increase in diet diversity category, HR: 0.72, 0.53–0.96; Figure 2) and the association remained after adjustments [adjusted HR (aHR): 0.46, 0.25–0.87; per increase in the diet diversity category, aHR: 0.67, 0.49–0.92; Figure 2]. High compared with low maternal diet diversity yielded aHRs of 0.75 for offspring IBD (0.55–1.02; Figure 3) and 0.81 for offspring CD (0.51–1.28; Figure 4). No association was found between maternal diet quality and the offspring’s risk of either IBD or its subtypes. The aHRs for high compared with low maternal diet quality were 0.90 (0.66–1.22; Figure 3) for IBD, 0.81 for CD (0.51–1.29; Figure 4), and 0.87 for UC in the offspring (0.47–1.60; Figure 2).

### Maternal intake of food groups

The prespecified analyses comparing high compared with maternal low intake of specified food groups did not show an association with offspring IBD or its subtypes. However, medium compared with low maternal dairy intake was associated with a higher risk of IBD (aHR: 1.42, 1.06–1.91; Supplemental Table 7) and CD in the offspring (aHR: 1.87, 1.18–2.97; Supplemental Table 8), but not UC (aHR: 1.11, 0.65–1.89; Supplemental Table 9). Compared with the lowest maternal intakes of fruits and whole grains, medium intakes were associated with a lower risk of offspring IBD (fruits, aHR: 0.69, 0.51–0.94; whole grains, aHR: 0.59, 0.43–0.83; Supplemental Table 7). We found no association between the maternal intake of any other food group and the offspring’s risk of IBD, CD, or UC, respectively (Supplemental Tables 7–9).

### Sensitivity analyses

High compared with low maternal diet diversity was associated with a lower risk of UC in the offspring when additionally adjusting for maternal antibiotics use in pregnancy (high compared with low, aHR: 0.46, 0.25–0.87; per increase in diet diversity category, aHR: 0.67, 0.49–0.92; Supplemental Table 10) and the child’s antibiotic use by 18 mo of age (high compared with low, aHR: 0.53, 0.28–1.01; per increase in diet diversity category, aHR: 0.72, 0.52–0.99; Supplemental Table 10). In line with the main analyses, analyses yielded essentially unchanged estimates for maternal diet diversity and the offspring’s UC risk when additionally adjusting for the child’s diet quality at 18 mo of age (aHR: 0.62, 0.30–1.28; Supplemental Table 11). Similar adjustments for analyses on high compared with low maternal diet diversity and quality and the offspring’s risk of IBD yielded aHRs that were close to our main analyses (Supplemental Table 12). Finally, analyses restricted to participants diagnosed with childhood-onset IBD (<18 y, *n* = 263) showed similar associations to those reported in the main analyses (Supplemental Table 13).

## Discussion

This nationwide pregnancy cohort study investigated maternal diet diversity, quality, and intakes of specific food groups in pregnancy and the risk of IBD in the offspring. In a cohort of >80,000 children with long-term follow-up, having a mother with a higher diet diversity in their pregnancy was associated with a significantly lower risk of UC in the offspring. Having a higher maternal diet quality or higher intakes of specific food groups in pregnancy was not associated with the offspring’s risk of subsequent IBD.

We conducted a population-based study with prospectively collected data, which improves the generalizability of our findings and the precision of our exposure assessments. We could conduct analyses of IBD subtypes by using extensive national registries and a large sample size. Using a well-validated algorithm to define IBD diagnosis [36], we limited the risk of capturing false cases. Another strength lies in the validated FFQ, specifically designed to assess the dietary intake of pregnant females in this cohort. Nevertheless, as in any self-reported dietary assessment tool, FFQ may not exactly estimate absolute dietary intakes [39], However, the FFQ used in this study is a valid method when ranking pregnant females on intakes of foods, nutrients, and energy [[22], [23], [24], [25]]. Importantly, we used the FFQ to capture the overall diet diversity and quality rather than the absolute intake of macro- or micronutrients. To improve the reliability of the exposure assessment, we excluded participants with implausible or unreliable dietary data. Furthermore, the generous background information allowed us to adjust for important confounders, including maternal educational level, smoking in pregnancy, and parental IBD [[40], [41], [42]]. We could also account for events later in life, such as the child’s early-life diet quality and antibiotic exposure.

MoBa recruited participants from all over Norway and collaborated with 50 of Norway’s 52 hospitals during the recruitment period [18]. However, females participating in MoBa were older, better educated, and less often smoked [43] than a representative sample of all Norwegian pregnant females at the time [44]. The self-selection for participation in MoBa could reduce the variation in the habitual diet in our sample and reduce differences in dietary exposures. Nevertheless, these differences have not affected exposure-outcome associations for other diseases in this cohort [44], Because of the study’s Nordic context, the results may primarily apply to other countries with comparable food culture, lifestyle, and IBD risk.

This study lacks biological data that prevented us from examining the potential mediating effects through the gut microbiota composition or examining if associations differed by genetic susceptibility to IBD. As with any observational study, we cannot rule out the possibility of unmeasured or residual confounding that may impede our ability to make causal inferences. Because of the explorative study design, we did not adjust the analyses for multiple comparisons [45]. We acknowledge that we did not assess maternal intake of ultraprocessed foods in pregnancy and its association with IBD in the offspring; however, it was outside the scope of this study. Although we adjusted our analyses for antibiotics in pregnancy and by the offspring’s age of 18 mo, we acknowledge that we were unable to adjust our analyses for antibiotics use during labor and in newborns specifically. Because our findings were based on a relatively small sample size of UC events, they should be corroborated in even larger populations. We encourage future studies to investigate the generalizability of our findings to other populations.

We observed that a high compared with low maternal diet diversity was associated with a 54% lower risk of UC in the child. Supporting the importance of diet diversity for a diverse gut microbiome [9], our findings suggest that the diversity of the maternal diet is independently associated with the child’s susceptibility to UC. Although attenuated, the association remained largely unchanged in sensitivity analyses adjusted for the child’s diet quality in early-life as well as maternal and child antibiotic use. However, we did not observe any association between maternal diet diversity and the offspring’s risk of CD. Although diet has been implicated as a risk factor for both CD and UC [6,46], UC has been suggested to be more prone to environmental triggers rather than genetic susceptibility [47].

Diet has been estimated to explain 20% of the variation in the gut microbiome [48], and the consumption of a diverse range of foods increases dietary fiber and other nutrient intakes favoring diversity in the gut microbiome composition [49]. Research has indicated that both the mother and neonate’s gut microbiome can be significantly influenced by food intake during pregnancy [50]. Consistent with the results of this study, a previous pregnancy cohort indicated that maternal diet diversity in pregnancy is associated with other immune-mediated diseases, including childhood atopy [51]. However, although previous studies have established an association between diet diversity and health outcomes in adults [52] as well as childhood conditions such as food allergy [49] and atopy [53], studies on maternal diet diversity in pregnancy and the offspring’s risk of IBD are scarce.

Several aspects of early-life diet have been associated with the risk of IBD, including the intake of specific food groups [5,6,10,35], and dietary patterns [3,4,10]. Our null findings on maternal diet quality and IBD risk are consistent with studies suggesting no association between high-quality diet in pregnancy and offspring atopic manifestations [54,55]. In contrast to our study, other studies have demonstrated a dose–response association between the intake of specific food groups and the risk of IBD in adults [[5], [6], [7]]. Although none of these studies have assessed maternal food intake in pregnancy, we cannot rule out the risk of chance affecting our results and we urge caution in interpreting our findings between maternal intake of food groups and offspring’s risk of IBD.

In this nationwide prospective cohort, a higher maternal diet diversity, rather than overall diet quality, was associated with a lower risk of UC in the offspring. These findings indicate that in-utero exposure to a high diet diversity may have durable implications for the offspring’s development of IBD and future studies should examine diet diversity at different ages and the risk of IBD.

## Author contributions

The authors’ responsibilities were as follows – AG, ALB, KM, KS: designed and conducted the study; AG: performed statistical analysis, interpreted the findings, and wrote the first draft of the manuscript; HI: provided statistical expertise; ALB, TCB, EMH, HI, KM, KS: interpreted the results, critically revised the manuscript, and provided intellectual content; AG, KM obtained funding; ALB, KM, KS: supervised the study; and all authors acknowledge full responsibility for the analyses and interpretation of data and have read and approved the final manuscript. KM, KS: have equal contributions and are the guarantors.

## Funding

Supported by The Swedish Society for Medical Research [S20-0007 to KM, TG-23-0002 to AG], The Swedish Research Council [2020-01980 to KM], ALF [ALFGBG-915661 to KM], and Henning and Johan Throne-Holst Foundation [000-00000 to AG]. The Norwegian Mother, Father, and Child Cohort Study receives support from the Norwegian Ministry of Health and Care Services and the Ministry of Education and Research. The funders had no role in study design, data collection, analysis, drafting of the manuscript, or the publication process.

## Data availability

All relevant data are included in the manuscript and its supplementary material. The lead authors (the manuscript’s guarantors) affirm that the manuscript is an honest, accurate, and transparent account of the study being reported; that no important aspects of the study have been omitted; and that any discrepancies from the study as planned (and, if relevant, registered) have been explained. The consent given by the participants does not open for storage of data on an individual level in repositories or journals. Researchers who want access to data sets for replication should apply through helsedata.no. Access to data sets requires approval from The Regional Committee for Medical and Health Research Ethics in Norway and an agreement with MoBa.

## Conflict of interest

The authors report no conflicts of interest.
