# Supplementary material for: Maternal diet in pregnancy and the risk of inflammatory bowel disease in the offspring: a prospective cohort study
Source: Am J Clin Nutr. 2024 Oct 24;121(1):32–9. doi: 10.1016/j.ajcnut.2024.10.017 (PMC11747187; doi:10.1016/j.ajcnut.2024.10.017)
Supplement: multimedia component 1 [file mmc1.docx]

**SUPPLEMENTARY TO**

*Maternal diet in pregnancy and the risk of inflammatory bowel disease in the offspring:
a prospective cohort study*

*Annie Guo*

TABLE OF CONTENT

[SUPPLEMENTARY INFORMATION 2](#_Toc161315653)

[SUPPLEMENTARY TABLES 3](#_Toc161315654)

[**Supplementary Table 1.** Description of the modified diet diversity score^a^ 3](#_Toc161315655)

[**Supplementary Table 2.** Description of the modified Prenatal Diet Quality Index used to assess maternal diet in pregnancy^a^ 4](#_Toc161315656)

[**Supplementary Table 3.** Definitions of food groups and included food items 5](#_Toc161315657)

[**Supplementary Table 4.** International Statistical Classification of Diseases and Health Related Problems, Tenth Revision (ICD-10) codes for inflammatory bowel disease^a^ 6](#_Toc161315658)

[**Supplementary Table 5.** Description of covariates considered in the adjusted analyses 7](#_Toc161315659)

[**Supplementary Table 6.** Follow-up time and incidence rate of inflammatory bowel disease, Crohn’s disease, and ulcerative colitis in the study cohort 8](#_Toc161315660)

[**Supplementary Table 7.** Maternal food intake in pregnancy and the offspring’s risk of inflammatory bowel disease 9](#_Toc161315661)

[**Supplementary Table 8.** Maternal food intake in pregnancy and the offspring’s risk of Crohn’s disease 11](#_Toc161315662)

[**Supplementary Table 9.** Maternal food intake in pregnancy and the offspring’s risk of ulcerative colitis 13](#_Toc161315663)

[**Supplementary Table 10.** Sensitivity analysis of maternal diet diversity in pregnancy and the child’s later risk of ulcerative colitis when additionally adjusting for the maternal use of antibiotics in pregnancy and the child’s antibiotic treatment by 18 months of age 15](#_Toc161315664)

[**Supplementary Table 11.** Sensitivity analysis of maternal diet diversity in pregnancy and the child’s later risk of inflammatory bowel disease and ulcerative colitis additionally adjusted for the child’s diet quality at 18 months (n=52,072) 16](#_Toc161315665)

[**Supplementary Table 12.** Sensitivity analysis of maternal diet diversity in pregnancy and the child’s later risk of inflammatory bowel disease when additionally adjusting for the maternal use of antibiotics in pregnancy and the child’s antibiotic treatment by 18 months of age 17](#_Toc161315666)

[**Supplementary Table 13.** Sensitivity analysis of maternal diet in pregnancy and inflammatory bowel disease restricting to childhood-onset <18 years (n=263) 18](#_Toc161315667)

[SUPPLEMENTARY FIGURES 20](#_Toc161315668)

[**Supplementary Figure 1.** Directed acyclic graph of potential confounders (red), mediators (grey), and ancestors (white) of IBD 20](#_Toc161315669)

[REFERENCES 21](#_Toc161315670)

# **SUPPLEMENTARY INFORMATION**

**The Norwegian Mother, Father, and Child Cohort Study**
The Norwegian Mother, Father, and Child Cohort Study (MoBa) was conducted in Norway between 1999 and 2008 [1]. It began in Bergen in 1999, where pregnant women were invited to join MoBa through mail prior to a routine ultrasound examination. By 2008, MoBa had gradually extended its reach to include 96% of all Norwegian hospitals with maternity units (n=50/52). All pregnant women residing in Norway were deemed eligible to participate. However, because all questionnaires and information were in Norwegian, the inability to read Norwegian was an exclusion criterion. The food frequency questionnaire used can be found on <https://www.fhi.no/en/ch/studies/moba/for-forskere-artikler/questionnaires-from-moba/> .

# **SUPPLEMENTARY TABLES**

## **Supplementary Table 1.** Description of the modified diet diversity index^1^

| **Food group** | **Food items (conversion factor)**^2^ | **Size of ¼ serving, g** |
| --- | --- | --- |
| Grains |  |  |
| Non-whole-grain breads | Low-fiber bread | 10 |
| Non-whole-grain cereals | Cornflakes | 13 |
| Non-whole-grain crackers | Crackers | 2 |
| Pasta | Spaghetti, pasta | 50 |
| Whole-grain breads | High-fiber bread | 11 |
| Whole-grain crispbread | High-fiber crispbread | 2.5 |
| Whole-grain cereals | High-fiber muesli and oatmeal porridge | 33 |
| Rice | Rice, couscous/millet | 43 |
| Vegetables |  |  |
| White potatoes | Mashed and fried potatoes, French fries | 30 |
| Nuts | Almonds, peanuts, other nuts | 13 |
| Legumes | Vegetable dishes with vegetables, legumes | 12 |
| Root vegetables | Rutabagas (swedes), carrots | 16 |
| Cruciferous vegetables | Cauliflower, broccoli, cabbage, Brussels sprouts | 15 |
| Leafy vegetables | Spinach, lettuce | 10 |
| Other vegetables | Alliums, mushrooms, peppers, cucumbers, celery, squash, corn, tomatoes, peas, avocados | 25 |
| Fruits |  |  |
| Citrus fruits, melons, berries | Oranges, grapefruit, all berries | 25 |
| All other fruits and juices | Apples, bananas, grapes, raisins, pears, mangoes, papayas, plums | 25 |
| Animal-based products |  |  |
| Red meat | Beef, pork, lamb, offal | 38–71 |
| Milk | Low-fat milk, yoghurt, pancakes and waffles (0.6) | 50 |
| Game | Reindeer, moose, wild boar | 38 |
| Poultry | Chicken, turkey | 38 |
| Cheese | Cheese, including brown and white cheese. | 5 |
| Eggs | Eggs, seagull eggs, pancakes (0.2), waffles (0.2) | 14 |
| Fish | Seafood, lean fish, fatty fish, roe, fish fingers (0.6) | 11 |
| Yoghurt | Yoghurt, probiotic milk | 31 |

^1^Originally developed by Borge et al. 2019 [2]. The dietary diversity score reflects a weighted average of scores for each of the four food subgroups. Each of the 4 broad food categories received a maximum diversity score of 2.5 of the 10 possible score points. Within each food subgroup, the score reflects the percentage of the possible maximum score. For example, a person consuming at least one-half serving in only 3 of 8 possible grain categories would receive a subgroup score of 3/8×2.5 or 0.94 of 2.5 points.

^2^For food items with several ingredients, we estimated the actual weight of the food item we were interested in, e.g., fish cake was calculated to consist of 60% lean fish.

## **Supplementary Table 2.** Description of the modified Prenatal Diet Quality Index^1^

| **Component** | **Food items (conversion factor)^2^** | **Recommended intake** | **Maximum score** |
| --- | --- | --- | --- |
| Fresh fruits and berries | Orange, plum, pear, banana, grapes, apple, peach, grapefruit, fruit salad, fruit spread, melon, papaya, other fruits, mango, strawberries and other berries, fruit juice (up to100g/day) | >250g/day | 10 |
| Vegetables | Carrots, cabbage, swede, pepper, celery, avocado, cauliflower, broccoli, other vegetables, olives, green mixed salad, lettuce, spinach, garlic, onion, frozen vegetables, peas, corn, Brussel sprouts, aubergine, squash, green beans, mushrooms, cucumber, tomato, vegetable spread, tomato juice (up to 100g/day) | >250g/day | 10 |
| Whole grain | High-fiber bread, crispbread, muesli, oatmeal porridge | ca 70g/day | 10 |
| Total fish | Tuna, mackerel, salmon, fish cake (0.6), fish fingers (0.6), fish casserole (0.3), pickled herring, sardines, cod, halibut, perch, other types of fish | 300-450g/day | 5 |
| Fatty fish | Mackerel, salmon, pickled herring, sardines | >200g/week (max 450g) | 5 |
| Red meat, including processed meat | Beef, pork, lamb, game, meatballs, hamburger, minced meat, sausage, beef, pork, hot dogs, salami, ham, roasted beef, cold cuts, bacon, offal | <500g/week (processed meat as low as possible) | 10 |
| Dairy | Yoghurt, low-fat and skimmed milk, cheese | 3 servings/day^3^ | 10 |
| Saturated fat | Total daily intake of saturated fat | <10E% | 10 |
| Trans fat | Total daily intake of trans-fat | <1E% | 10 |
| Salt | Total daily intake of salt | <5g/day (=2g sodium/day) | 10 |
| Added sugar | Total daily intake of added sugar | <10E% | 10 |
| Dietary Diversity | Diversity of foods within *Grains, vegetables, fruit,* and *animal products* (see Supplementary Table 5) | >¼ serving/day of each food items yields a positive score | 10 |

E%=percentage of total daily energy intake.

^1^Components with a minimum intake recommendation (fresh fruits and berries, vegetables, whole grain, dairy) were calculated: reported intake/recommended intake * 10. Participants reporting below the recommended intake were given a proportionately lower/less score, e.g., participants reporting half the intake received 5 scores, while intakes above the recommended were given the maximum score of 10.
Components with a maximum recommended intake (red meat, saturated fat, trans fat, salt, added sugar) were calculated: recommended intake/reported intake * 10. Participants reporting intakes above the recommended intake were given less score, e.g., participants reporting twice the amount of the intake received 5 scores, while intakes less than the recommended were given the maximum score of 10.
Components with a recommended range with a minimum and maximum intake recommendation (total fish, fatty fish) were calculated using the formula for minimum and maximum intakes. Formula for minimum intake recommendation was used if the reported intake was lower than the recommended intake. Formula for maximum intake recommendation was used if the reported intake was above the upper recommended intake. Only participants reporting within the recommended intake range were given the maximum score of 10. The maximum total index was 110 [2, 3].

^2^For food items with several ingredients, we estimated the actual weight of the food item we were interested in, e.g., fish cake was calculated to consist of 60% lean fish.

^3^1 serving = 20 g of cheese, 2 dl (200g) of milk or 1 serving (125 g) of yogurt. Recommended intake of foremost low-fat dairy products (excluding, e.g., blue cheese).

## **Supplementary Table 3.** Definitions of food groups and included food items

| **Food groups** | **Food items (conversion factor)^1^** |
| --- | --- |
| Red meat | Low-and high-fat cold cuts, salami, liver paste, sausage, beef, pork, hot dogs, meatballs, hamburger, minced meat, steak, pork, spareribs, bacon, lamb, game, liver, black pudding, meat in composite dishes; minced meat in stew (0.3), pasta with meat (0.3), beef stew (0.5), pork stew (0.35), lamb and game stew (0.4) |
| White meat | All types of poultry, including turkey, chicken, game (grouse), other poultry |
| Fatty fish | Roe, sardines, salmon, herring, mackerel, fish liver |
| Lean fish and seafood | Shrimps, crab, tuna, cod, halibut, perch, mussels, crab, fish in composite dishes; fish cake (0.6), fish fingers (0.6), fish casserole (0.3) |
| Dairy | All types of dairy products, including milk, yoghurt, cheese, cream, butter, diary desserts, |
| Fruits | All types of fruit, including fresh fruits, fruit salad, berries and fruit spread |
| Vegetables | All types of vegetables, including raw, cooked and frozen vegetables, mushrooms, legumes, olives, and pasta with vegetables (0.4) |
| Whole grains | Bread (dark/fibre), crispbread, muesli (unsweetened/sweetened), oatmeal porridge |
| Refined grains | Bread (white/whole meal), crackers, cornflakes, pizza, pancakes, spaghetti, rice, millet, waffle |
| Salty foods | French fries, potato chips, salty snacks |
| Sugary foods and drinks | Jam, honey, sweetened nut and chocolate spread, table sugar, chocolate milk, juice with nectar, sugar-sweetened soft drinks, milk pudding, ice cream, sorbet, vanilla sauce, bun, Danish pastry, doughnut, cake, cookies, chocolate, candies, sweets, marzipan |

^1^For food items with several ingredients, we estimated the actual weight of the food item we were interested in, e.g., fish cake was calculated to consist of 60% lean fish.

##

## **Supplementary Table 4.** International Statistical Classification of Diseases and Health Related Problems, Tenth Revision (ICD-10) codes for inflammatory bowel disease^1^

| **Disease** | **ICD-10 (1997–)** |
| --- | --- |
| Inflammatory bowel disease | K52.3, CD + UC or mixed diagnosis of CD, UC and IBD-U |
| Crohn’s disease | K50 |
| Ulcerative colitis | K51 |
| IBD-unclassified | K52.3 |

CD=Crohn’s disease; IBD=inflammatory bowel disease; IBD-U=inflammatory bowel disease unclassified; UC=ulcerative colitis.

^1^IBD diagnosis was defined as ≥2 inpatient or non-primary outpatient care visits with an ICD-10 code for IBD [4, 5]. Patients who shifted between subtypes of IBD but only had one diagnosis in the past 5 years were classified according to their most recent diagnosis. Patients with several CD, UC, or IBD-U codes during follow-up were classified as (“any, non-specific”) IBD.

## **Supplementary Table 5.** Description of covariates considered in the adjusted analyses

| **Covariates** | **Data source** | **Description** |
| --- | --- | --- |
| Child’s sex | Medical Birth Registry of Norway | Female/male |
| Parental IBD | Norwegian Patient Registry | Defined as >1 parent with IBD |
| Maternal origin | 15 GW questionnaire | Mother’s native language |
| Maternal educational level | 15 GW questionnaire | <11, 12, >13 years of education |
| Maternal comorbidities | 15 GW questionnaire | Type 1 diabetes, rheumatoid arthritis and/or thyroid disease |
| Maternal pre-pregnant BMI | 15 GW questionnaire | Based on the weight three months prior to pregnancy, kg/m^2^ |
| Maternal antibiotic use in pregnancy | 15 GW, 30 GW, 6 months questionnaires | Yes/no |
| Child antibiotic use at age 18 months | 18 months questionnaire | Yes/no |
| Child diet quality at age 18 months^1^ | 18 months questionnaire | Low, medium, and high level where a high diet quality represents a higher intake of fruits and vegetables, fish, and dairy foods and lower intakes of meat, soft drinks, and salty and sweet snacks |

BMI=body mass index; IBD=inflammatory bowel disease; GW=gestational week.

^1^Further information is provided elsewhere [6].

## **Supplementary Table 6.** Follow-up time and incidence rate of inflammatory bowel disease, Crohn’s disease, and ulcerative colitis in the study cohort

|  | n event | PY of follow-up | Incidence rate per 100,000 PY (95%CI) | Cumulative incidence at the end of data capture^1^ |
| --- | --- | --- | --- | --- |
| Inflammatory bowel disease | 268 | 1,367,837 | 19.6 (17.3 to 22.1) | 0.24% (0.21 to 0.27%) |
| Crohn’s disease | 119 | 1,368,481 | 8.7 (7.2 to 10.4) | 0.11% (0.09 to 0.13%) |
| Ulcerative colitis | 76 | 1,368,640 | 5.6 (4.4 to 7.0) | 0.07% (0.05 to 0.09%) |

CI=confidence interval; PY=person-years. Incidence rates were estimated using Poisson regression. The cumulative incidence was estimated using the Kaplan–Meier product limit estimator to account for censoring.

^1^December 31, 2021.

**Supplementary Table 7.** Maternal intakes of specific food groups in pregnancy and the offspring’s risk of inflammatory bowel disease

|  |  | | | **Unadjusted** | | **Adjusted** | |
| --- | --- | --- | --- | --- | --- | --- | --- |
| **Diet exposure** | **Incidence rate (95% CI)**  **per 100,000 PY** | **N** | **n event** | **HR** | **95% CI** | **HR^1^** | **95% CI** |
| Red meat |  |  |  |  |  |  |  |
| Low | 16.8 (13.2 to 21.0) | 28,297 | 75 | Ref. | Ref. | Ref. | Ref. |
| Medium | 21.7 (17.6 to 26.4) | 28,416 | 99 | 1.25 | 0.93 to 1.69 | 1.30 | 0.95 to 1.77 |
| High | 20.3 (16.4 to 24.8) | 28,416 | 94 | 1.14 | 0.84 to 1.54 | 1.15 | 0.84 to 1.58 |
| Per increase in category | |  |  | 1.06 | 0.92 to 1.22 | 1.06 | 0.92 to 1.23 |
| White meat |  |  |  |  |  |  |  |
| Low | 23.1 (19.0 to 27.9) | 28,298 | 108 | Ref. | Ref. | Ref. | Ref. |
| Medium | 16.7 (13.1 to 20.9) | 28,415 | 76 | 0.75 | 0.56 to 1.01 | 0.74 | 0.54 to 1.00 |
| High | 18.9 (15.1 to 23.4) | 28,416 | 84 | 0.90 | 0.67 to 1.19 | 0.89 | 0.66 to 1.20 |
| Per increase in category | |  |  | 0.94 | 0.80 to 1.09 | 0.94 | 0.80 to 1.10 |
| Fatty fish |  |  |  |  |  |  |  |
| Low | 20.0 (16.1 to 24.5) | 28,297 | 92 | Ref. | Ref. | Ref. | Ref. |
| Medium | 18.7 (14.9 to 23.1) | 28,417 | 85 | 0.96 | 0.71 to 1.29 | 1.00 | 0.73 to 1.36 |
| High | 20.1 (16.2 to 24.7) | 28,415 | 91 | 1.04 | 0.78 to 1.39 | 1.10 | 0.82 to 1.49 |
| Per increase in category | |  |  | 1.02 | 0.88 to 1.18 | 1.05 | 0.90 to 1.22 |
| Lean fish and seafood |  |  |  |  |  |  |  |
| Low | 22.0 (17.9 to 26.7) | 28,297 | 99 | Ref. | Ref. | Ref. | Ref. |
| Medium | 16.7 (13.2 to 21.0) | 28,416 | 76 | 0.76 | 0.56 to 1.02 | 0.79 | 0.58 to 1.07 |
| High | 20.1 (16.2 to 24.6) | 28,416 | 93 | 0.88 | 0.66 to 1.16 | 0.91 | 0.68 to 1.22 |
| Per increase in category | |  |  | 0.93 | 0.80 to 1.09 | 0.95 | 0.82 to 1.11 |
| Dairy |  |  |  |  |  |  |  |
| Low | 17.4 (13.8 to 21.7) | 28,297 | 79 | Ref. | Ref. | Ref. | Ref. |
| Medium | 24.1 (19.8 to 29.0) | 28,416 | 110 | 1.38 | 1.03 to 1.84 | 1.42 | 1.06 to 1.91 |
| High | 17.3 (13.7 to 21.5) | 28,416 | 79 | 0.98 | 0.72 to 1.35 | 1.00 | 0.72 to 1.38 |
| Per increase in category | |  |  | 0.99 | 0.86 to 1.14 | 1.00 | 0.87 to 1.15 |
| Fruits |  |  |  |  |  |  |  |
| Low | 23.3 (19.1 to 28.2) | 28,297 | 107 | Ref. | Ref. | Ref. | Ref. |
| Medium | 15.4 (12.0 to 19.4) | 28,416 | 70 | 0.68 | 0.50 to 0.91 | 0.69 | 0.51 to 0.94 |
| High | 20.1 (16.1 to 24.6) | 28,416 | 91 | 0.89 | 0.67 to 1.17 | 0.90 | 0.67 to 1.21 |
| Per increase in category | |  |  | 0.93 | 0.80 to 1.09 | 0.94 | 0.80 to 1.11 |
| Vegetables |  |  |  |  |  |  |  |
| Low | 20.7 (16.7 to 25.3) | 28,297 | 95 | Ref. | Ref. | Ref. | Ref. |
| Medium | 17.1 (13.5 to 21.3) | 28,416 | 78 | 0.84 | 0.62 to 1.13 | 0.81 | 0.60 to 1.10 |
| High | 21.0 (17.0 to 25.7) | 28,416 | 95 | 1.05 | 0.79 to 1.40 | 0.98 | 0.73 to 1.32 |
| Per increase in category | |  |  | 1.02 | 0.88 to 1.19 | 1.00 | 0.85 to 1.17 |
| Whole grains |  |  |  |  |  |  |  |
| Low | 24.1 (19.9 to 29.0) | 28,327 | 112 | Ref. | Ref. | Ref. | Ref. |
| Medium | 13.5 (10.3 to 17.3) | 28,387 | 61 | 0.59 | 0.43 to 0.81 | 0.59 | 0.43 to 0.83 |
| High | 21.1 (17.0 to 25.7) | 28,415 | 95 | 0.92 | 0.70 to 1.22 | 0.99 | 0.74 to 1.32 |
| Per increase in category | |  |  | 0.95 | 0.81 to 1.11 | 0.99 | 0.84 to 1.17 |
| Refined grains |  |  |  |  |  |  |  |
| Low | 21.2 (17.2 to 26.0) | 28,297 | 95 | Ref. | Ref. | Ref. | Ref. |
| Medium | 18.0 (14.3 to 22.3) | 28,417 | 82 | 0.83 | 0.61 to 1.11 | 0.77 | 0.57 to 1.05 |
| High | 19.6 (15.8 to 24.0) | 28,415 | 91 | 0.87 | 0.65 to 1.16 | 0.85 | 0.63 to 1.15 |
| Per increase in category | |  |  | 0.93 | 0.80 to 1.08 | 0.92 | 0.78 to 1.08 |
| Fat-and salt-dense food |  |  |  |  |  |  |  |
| Low | 20.0 (16.1 to 24.5) | 28,754 | 92 | Ref. | Ref. | Ref. | Ref. |
| Medium | 17.5 (13.9 to 21.8) | 29,126 | 82 | 0.87 | 0.64 to 1.18 | 0.79 | 0.58 to 1.08 |
| High | 21.4 (17.3 to 26.1) | 27,249 | 94 | 1.05 | 0.78 to 1.40 | 0.97 | 0.72 to 1.30 |
| Per increase in category | |  |  | 1.02 | 0.88 to 1.19 | 0.98 | 0.84 to 1.15 |
| Sugary foods and drinks |  |  |  |  |  |  |  |
| Low | 17.8 (14.2 to 22.2) | 28,297 | 80 | Ref. | Ref. | Ref. | Ref. |
| Medium | 17.9 (14.2 to 22.2) | 28,416 | 81 | 0.99 | 0.73 to 1.35 | 0.96 | 0.70 to 1.32 |
| High | 22.9 (18.8 to 27.7) | 28,416 | 107 | 1.21 | 0.90 to 1.62 | 1.19 | 0.88 to 1.61 |
| Per increase in category | |  |  | 1.10 | 0.95 to 1.28 | 1.10 | 0.94 to 1.28 |

CI=confidence interval; HR=hazard ratio; PY=person-years. Incidence rates were estimated using Poisson regression. Hazard ratios were estimated using Cox proportional hazards regression.

^1^Adjusted for the child’s sex, parental inflammatory bowel disease, and mother’s origin, education level, comorbidities, and pre-pregnancy body mass index (Supplementary Table 5).

**Supplementary Table 8.** Maternal intakes of specific food groups in pregnancy and the offspring’s risk of Crohn’s disease

|  | **Unadjusted** | | | | | **Adjusted** | |
| --- | --- | --- | --- | --- | --- | --- | --- |
| **Diet exposure** | **Incidence rate (95% CI) per 100,000 PY** | **N** | **n event** | **HR** | **95% CI** | **HR^1^** | **95% CI** |
| Red meat |  |  |  |  |  |  |  |
| Low | 6.9 (4.7 to 9.8) | 28,297 | 31 | Ref. | Ref. | Ref. | Ref. |
| Medium | 10.3 (7.6 to 13.7) | 28,416 | 47 | 1.45 | 0.93 to 2.26 | 1.51 | 0.95 to 2.40 |
| High | 8.8 (6.3 to 12.0) | 28,416 | 41 | 1.22 | 0.76 to 1.94 | 1.19 | 0.73 to 1.95 |
| Per increase in category | |  |  | 1.09 | 0.88 to 1.34 | 1.07 | 0.86 to 1.34 |
| White meat |  |  |  |  |  |  |  |
| Low | 10.1 (7.4 to 13.4) | 28,298 | 47 | Ref. | Ref. | Ref. | Ref. |
| Medium | 7.9 (5.5 to 10.9) | 28,415 | 36 | 0.81 | 0.53 to 1.25 | 0.83 | 0.53 to 1.30 |
| High | 8.1 (5.7 to 11.2) | 28,416 | 36 | 0.86 | 0.56 to 1.33 | 0.90 | 0.58 to 1.42 |
| Per increase in category | |  |  | 0.92 | 0.74 to 1.16 | 0.95 | 0.75 to 1.20 |
| Fatty fish |  |  |  |  |  |  |  |
| Low | 9.1 (6.6 to 12.3) | 28,297 | 42 | Ref. | Ref. | Ref. | Ref. |
| Medium | 8.6 (6.1 to 11.7) | 28,417 | 39 | 0.96 | 0.62 to 1.49 | 1.02 | 0.64 to 1.62 |
| High | 8.4 (5.9 to 11.5) | 28,415 | 38 | 0.94 | 0.61 to 1.47 | 1.05 | 0.66 to 1.66 |
| Per increase in category | |  |  | 0.97 | 0.78 to 1.21 | 1.02 | 0.81 to 1.29 |
| Lean fish and seafood |  |  |  |  |  |  |  |
| Low | 9.5 (6.9 to 12.8) | 28,297 | 43 | Ref. | Ref. | Ref. | Ref. |
| Medium | 7.5 (5.2 to 10.5) | 28,416 | 34 | 0.78 | 0.50 to 1.23 | 0.80 | 0.50 to 1.28 |
| High | 9.1 (6.5 to 12.2) | 28,416 | 42 | 0.92 | 0.60 to 1.41 | 0.97 | 0.62 to 1.51 |
| Per increase in category | |  |  | 0.96 | 0.76 to 1.20 | 0.98 | 0.78 to 1.25 |
| Dairy |  |  |  |  |  |  |  |
| Low | 6.4 (4.3 to 9.2) | 28,297 | 29 | Ref. | Ref. | Ref. | Ref. |
| Medium | 11.6 (8.7 to 15.2) | 28,416 | 53 | 1.81 | 1.16 to 2.83 | 1.87 | 1.18 to 2.97 |
| High | 8.1 (5.7 to 11.1) | 28,416 | 37 | 1.25 | 0.77 to 2.04 | 1.27 | 0.77 to 2.10 |
| Per increase in category | |  |  | 1.10 | 0.90 to 1.34 | 1.10 | 0.90 to 1.35 |
| Fruits |  |  |  |  |  |  |  |
| Low | 10.7 (7.9 to 14.1) | 28,297 | 49 | Ref. | Ref. | Ref. | Ref. |
| Medium | 7.0 (4.8 to 9.9) | 28,416 | 32 | 0.67 | 0.43 to 1.05 | 0.72 | 0.45 to 1.13 |
| High | 8.4 (5.9 to 11.5) | 28,416 | 38 | 0.80 | 0.52 to 1.23 | 0.86 | 0.55 to 1.34 |
| Per increase in category | |  |  | 0.89 | 0.70 to 1.12 | 0.92 | 0.73 to 1.17 |
| Vegetables |  |  |  |  |  |  |  |
| Low | 8.7 (6.2 to 11.9) | 28,297 | 40 | Ref. | Ref. | Ref. | Ref. |
| Medium | 8.1 (5.7 to 11.2) | 28,416 | 37 | 0.94 | 0.61 to 1.47 | 0.91 | 0.57 to 1.43 |
| High | 9.3 (6.7 to 12.5) | 28,416 | 42 | 1.09 | 0.71 to 1.69 | 1.00 | 0.64 to 1.58 |
| Per increase in category | |  |  | 1.05 | 0.84 to 1.31 | 1.00 | 0.79 to 1.27 |
| Whole grains |  |  |  |  |  |  |  |
| Low | 11.0 (8.2 to 14.4) | 28,327 | 51 | Ref. | Ref. | Ref. | Ref. |
| Medium | 6.4 (4.3 to 9.2) | 28,387 | 29 | 0.61 | 0.38 to 0.96 | 0.64 | 0.40 to 1.03 |
| High | 8.6 (6.1 to 11.8) | 28,415 | 39 | 0.82 | 0.54 to 1.25 | 0.88 | 0.57 to 1.37 |
| Per increase in category | |  |  | 0.89 | 0.70 to 1.13 | 0.93 | 0.73 to 1.19 |
| Refined grains |  |  |  |  |  |  |  |
| Low | 9.8 (7.1 to 13.2) | 28,297 | 44 | Ref. | Ref. | Ref. | Ref. |
| Medium | 7.2 (5.0 to 10.2) | 28,417 | 33 | 0.72 | 0.46 to 1.13 | 0.68 | 0.42 to 1.09 |
| High | 9.0 (6.5 to 12.2) | 28,415 | 42 | 0.88 | 0.57 to 1.35 | 0.88 | 0.56 to 1.38 |
| Per increase in category | |  |  | 0.93 | 0.74 to 1.18 | 0.93 | 0.73 to 1.20 |
| Fat-and salt-dense food |  |  |  |  |  |  |  |
| Low | 9.6 (6.9 to 12.8) | 28,754 | 44 | Ref. | Ref. | Ref. | Ref. |
| Medium | 6.4 (4.3 to 9.2) | 29,126 | 30 | 0.67 | 0.42 to 1.06 | 0.62 | 0.38 to 0.99 |
| High | 10.2 (7.5 to 13.7) | 27,249 | 45 | 1.05 | 0.69 to 1.60 | 0.94 | 0.61 to 1.44 |
| Per increase in category | |  |  | 1.03 | 0.81 to 1.30 | 0.96 | 0.75 to 1.23 |
| Sugary foods and drinks |  |  |  |  |  |  |  |
| Low | 8.0 (5.6 to 11.1) | 28,297 | 36 | Ref. | Ref. | Ref. | Ref. |
| Medium | 8.6 (6.1 to 11.8) | 28,416 | 39 | 1.06 | 0.67 to 1.68 | 1.01 | 0.63 to 1.62 |
| High | 9.4 (6.9 to 12.7) | 28,416 | 44 | 1.12 | 0.71 to 1.75 | 1.09 | 0.69 to 1.74 |
| Per increase in category | |  |  | 1.06 | 0.85 to 1.32 | 1.05 | 0.83 to 1.32 |

CI=confidence interval; HR=hazard ratio; PY=person-years. Incidence rates were estimated using Poisson regression. Hazard ratios were estimated using Cox proportional hazards regression.

^1^Adjusted for the child’s sex, parental inflammatory bowel disease, and mother’s origin, education level, comorbidities, and pre-pregnancy body mass index (Supplementary Table 5).

**Supplementary Table 9.** Maternal intakes of specific food groups in pregnancy and the offspring’s risk of ulcerative colitis

|  | **Unadjusted** | | | | | **Adjusted** | |
| --- | --- | --- | --- | --- | --- | --- | --- |
| **Diet exposure** | **Incidence rate (95% CI)**  **per 100,000 PY** | **N** | **n event** | **HR** | **95% CI** | **HR^1^** | **95% CI** |
| Red meat |  |  |  |  |  |  |  |
| Low | 4.2 (2.6 to 6.6) | 28,297 | 19 | Ref. | Ref. | Ref. | Ref. |
| Medium | 6.8 (4.6 to 9.6) | 28,416 | 31 | 1.52 | 0.86 to 2.68 | 1.63 | 0.90 to 2.98 |
| High | 5.6 (3.7 to 8.2) | 28,416 | 26 | 1.20 | 0.66 to 2.17 | 1.23 | 0.66 to 2.31 |
| Per increase in category | |  |  | 1.08 | 0.83 to 1.40 | 1.08 | 0.83 to 1.42 |
| White meat |  |  |  |  |  |  |  |
| Low | 5.6 (3.6 to 8.2) | 28,298 | 26 | Ref. | Ref. | Ref. | Ref. |
| Medium | 4.4 (2.7 to 6.8) | 28,415 | 20 | 0.85 | 0.48 to 1.52 | 0.72 | 0.39 to 1.33 |
| High | 6.7 (4.5 to 9.6) | 28,416 | 30 | 1.41 | 0.83 to 2.40 | 1.32 | 0.76 to 2.27 |
| Per increase in category | |  |  | 1.20 | 0.89 to 1.60 | 1.15 | 0.85 to 1.57 |
| Fatty fish |  |  |  |  |  |  |  |
| Low | 5.4 (3.5 to 8.0) | 28,297 | 25 | Ref. | Ref. | Ref. | Ref. |
| Medium | 5.7 (3.7 to 8.4) | 28,417 | 26 | 1.10 | 0.64 to 1.91 | 1.14 | 0.65 to 2.01 |
| High | 5.5 (3.6 to 8.1) | 28,415 | 25 | 1.07 | 0.62 to 1.85 | 1.07 | 0.61 to 1.89 |
| Per increase in category | |  |  | 1.04 | 0.79 to 1.35 | 1.03 | 0.79 to 1.36 |
| Lean fish and seafood |  |  |  |  |  |  |  |
| Low | 6.7 (4.5 to 9.5) | 28,297 | 30 | Ref. | Ref. | Ref. | Ref. |
| Medium | 5.3 (3.4 to 7.9) | 28,416 | 24 | 0.79 | 0.47 to 1.33 | 0.83 | 0.48 to 1.43 |
| High | 4.7 (3.0 to 7.2) | 28,416 | 22 | 0.67 | 0.39 to 1.15 | 0.69 | 0.39 to 1.23 |
| Per increase in category | |  |  | 0.81 | 0.62 to 1.07 | 0.83 | 0.63 to 1.11 |
| Dairy |  |  |  |  |  |  |  |
| Low | 6.6 (4.5 to 9.4) | 28,297 | 30 | Ref. | Ref. | Ref. | Ref. |
| Medium | 6.8 (4.6 to 9.6) | 28,416 | 31 | 1.02 | 0.61 to 1.70 | 1.11 | 0.65 to 1.89 |
| High | 3.3 (1.8 to 5.4) | 28,416 | 15 | 0.49 | 0.26 to 0.92 | 0.51 | 0.27 to 0.99 |
| Per increase in category | |  |  | 0.73 | 0.56 to 0.96 | 0.75 | 0.57 to 0.99 |
| Fruits |  |  |  |  |  |  |  |
| Low | 7.2 (4.9 to 10.1) | 28,297 | 33 | Ref. | Ref. | Ref. | Ref. |
| Medium | 4.0 (2.3 to 6.2) | 28,416 | 18 | 0.57 | 0.32 to 1.02 | 0.52 | 0.29 to 0.95 |
| High | 5.5 (3.6 to 8.1) | 28,416 | 25 | 0.80 | 0.48 to 1.35 | 0.76 | 0.44 to 1.31 |
| Per increase in category | |  |  | 0.88 | 0.65 to 1.18 | 0.85 | 0.62 to 1.16 |
| Vegetables |  |  |  |  |  |  |  |
| Low | 6.8 (4.6 to 9.6) | 28,297 | 31 | Ref. | Ref. | Ref. | Ref. |
| Medium | 3.9 (2.3 to 6.2) | 28,416 | 18 | 0.60 | 0.34 to 1.07 | 0.57 | 0.31 to 1.03 |
| High | 6.0 (3.9 to 8.7) | 28,416 | 27 | 0.93 | 0.55 to 1.58 | 0.79 | 0.45 to 1.39 |
| Per increase in category | |  |  | 0.96 | 0.71 to 1.29 | 0.87 | 0.63 to 1.20 |
| Whole grains |  |  |  |  |  |  |  |
| Low | 6.0 (4.0 to 8.7) | 28,327 | 28 | Ref. | Ref. | Ref. | Ref. |
| Medium | 4.2 (2.5 to 6.6) | 28,387 | 19 | 0.75 | 0.41 to 1.38 | 0.68 | 0.35 to 1.31 |
| High | 6.4 (4.3 to 9.2) | 28,415 | 29 | 1.17 | 0.69 to 1.98 | 1.25 | 0.72 to 2.17 |
| Per increase in category | |  |  | 1.08 | 0.81 to 1.45 | 1.13 | 0.82 to 1.54 |
| Refined grains |  |  |  |  |  |  |  |
| Low | 6.0 (4.0 to 8.8) | 28,297 | 27 | Ref. | Ref. | Ref. | Ref. |
| Medium | 4.8 (3.0 to 7.3) | 28,417 | 22 | 0.77 | 0.43 to 1.36 | 0.69 | 0.38 to 1.26 |
| High | 5.8 (3.8 to 8.4) | 28,415 | 27 | 0.87 | 0.50 to 1.51 | 0.87 | 0.49 to 1.54 |
| Per increase in category | |  |  | 0.93 | 0.70 to 1.25 | 0.93 | 0.68 to 1.28 |
| Fat-and salt-dense food |  |  |  |  |  |  |  |
| Low | 6.7 (4.6 to 9.6) | 28,754 | 31 | Ref. | Ref. | Ref. | Ref. |
| Medium | 4.7 (2.9 to 7.1) | 29,126 | 22 | 0.69 | 0.40 to 1.20 | 0.57 | 0.31 to 1.02 |
| High | 5.2 (3.3 to 7.8) | 27,249 | 23 | 0.75 | 0.44 to 1.30 | 0.72 | 0.41 to 1.27 |
| Per increase in category | |  |  | 0.86 | 0.64 to 1.15 | 0.83 | 0.60 to 1.14 |
| Sugary foods and drinks |  |  |  |  |  |  |  |
| Low | 4.9 (3.1 to 7.4) | 28,297 | 22 | Ref. | Ref. | Ref. | Ref. |
| Medium | 5.3 (3.4 to 7.9) | 28,416 | 24 | 1.06 | 0.60 to 1.86 | 1.11 | 0.62 to 2.00 |
| High | 6.4 (4.3 to 9.2) | 28,416 | 30 | 1.18 | 0.67 to 2.07 | 1.22 | 0.67 to 2.21 |
| Per increase in category | |  |  | 1.09 | 0.82 to 1.44 | 1.10 | 0.82 to 1.48 |

CI=confidence interval; HR=hazard ratio; PY=person-years. Incidence rates were estimated using Poisson regression. Hazard ratios were estimated using Cox proportional hazards regression.

^1^Adjusted for the child’s sex, parental inflammatory bowel disease, and mother’s origin, education level, comorbidities, and pre-pregnancy body mass index (Supplementary Table 5).

**Supplementary Table 10.** Sensitivity analysis of maternal diet diversity in pregnancy and the offspring’s risk of ulcerative colitis when additionally adjusting for the maternal antibiotic use in pregnancy and the child’s antibiotic use by 18 months of age

|  | **Ulcerative colitis** | | | | | | | |
| --- | --- | --- | --- | --- | --- | --- | --- | --- |
|  | **Adjusted model 1^1^** | | | | **Adjusted model 2^2^** | | | |
|  | **N** | **n event** | **HR** | **95% CI** | **N** | **n event** | **HR** | **95% CI** |
| Diet quality in pregnancy | | |  |  |  |  |  |  |
| Low | 27,493 | 26 | Ref. | Ref. | 24,845 | 23 | Ref. | Ref. |
| Medium | 27,568 | 25 | 1.01 | 0.58 to 1.75 | 25,207 | 22 | 0.99 | 0.55 to 1.78 |
| High | 27,566 | 20 | 0.87 | 0.47 to 1.61 | 25,170 | 20 | 0.97 | 0.52 to 1.84 |
| Per increase in category | | | 0.93 | 0.69 to 1.26 |  |  | 0.99 | 0.72 to 1.35 |
| Diet diversity in pregnancy | | |  |  |  |  |  |  |
| Low | 27,757 | 35 | Ref. | Ref. | 25,289 | 31 | Ref. | Ref. |
| Medium | 28,762 | 22 | 0.63 | 0.37 to 1.09 | 26,222 | 20 | 0.65 | 0.36 to 1.15 |
| High | 26,108 | 14 | 0.46 | 0.25 to 0.87 | 23,711 | 14 | 0.53 | 0.28 to 1.01 |
| Per increase in category | | | 0.67 | 0.49 to 0.92 |  |  | 0.72 | 0.52 to 0.99 |

CI=confidence interval; HR=hazard ratio. Hazard ratios were estimated using Cox proportional hazards regression.

^1^Adjusted for child’s sex, parental inflammatory bowel disease, mother’s origin, education level, comorbidities, pre-pregnancy body mass index, and the maternal use of antibiotics in pregnancy (Supplementary Table 5). The analysis includes a subgroup of mother-child pairs with valid data on maternal diet and maternal antibiotic use in pregnancy (n=82,627).

^2^Adjusted for child’s sex, parental inflammatory bowel disease, and mother’s origin, education level, comorbidities, pre-pregnancy body mass index, and the child’s antibiotic treatment by 18 months of age (Supplementary Table 5). The analysis includes a subgroup of mother-child pairs with valid data on maternal diet and child’s antibiotic use by 18 months of age (n=75,222).

**Supplementary Table 11.** Sensitivity analysis of maternal diet diversity in pregnancy and the offspring’s risk of inflammatory bowel disease and ulcerative colitis additionally adjusted for the child’s diet quality by 18 months of age (n=52,072)^1^

|  |  |  | **Inflammatory bowel disease** | | **Ulcerative colitis** | |
| --- | --- | --- | --- | --- | --- | --- |
|  | **N** | **n event** | **aHR** | **95% CI** | **aHR** | **95% CI** |
| Diet diversity in pregnancy | | |  |  |  |  |
| Low | 17,573 | 20 | Ref. | Ref. | Ref. | Ref. |
| Medium | 17,973 | 17 | 0.97 | 0.68 to 1.37 | 0.85 | 0.45 to 1.62 |
| High | 16,526 | 11 | 0.90 | 0.62 to 1.31 | 0.62 | 0.30 to 1.28 |
| Per increase in category | |  | 0.95 | 0.79 to 1.14 | 0.79 | 0.56 to 1.12 |

CI=confidence interval; HR=hazard ratio. Hazard ratios were estimated using Cox proportional hazards regression.

^1^Adjusted for the child’s sex and diet quality at 18 months of age, parental inflammatory bowel disease, as well as the mother’s origin, education level, comorbidities, pre-pregnancy body mass index, and the child’s diet quality by 18 months of age (Supplementary Table 5).

**Supplementary Table 12.** Sensitivity analysis of maternal diet diversity in pregnancy and the offspring’s risk of inflammatory bowel disease when additionally adjusting for the maternal antibiotic use in pregnancy and the child’s antibiotic use by 18 months of age

|  | **Inflammatory bowel disease** | | | | | | | |
| --- | --- | --- | --- | --- | --- | --- | --- | --- |
|  | **Adjusted model 1**^1^ | | | | **Adjusted model 2**^2^ | | | |
|  | **N** | **n event** | **HR** | **95% CI** | **N** | **n event** | **HR** | **95% CI** |
| Diet quality in pregnancy | | |  |  |  |  |  |  |
| Low | 27,493 | 96 | Ref. | Ref. | 24,845 | 85 | Ref. | Ref. |
| Medium | 27,568 | 81 | 0.89 | 0.66 to 1.19 | 25,207 | 75 | 0.91 | 0.67 to 1.24 |
| High | 27,566 | 78 | 0.90 | 0.66 to 1.22 | 25,170 | 74 | 0.95 | 0.69 to 1.30 |
| Per increase in category | | | 0.95 | 0.81 to 1.11 |  |  | 0.97 | 0.83 to 1.14 |
| Diet diversity in pregnancy | | |  |  |  |  |  |  |
| Low | 27,757 | 104 | Ref. | Ref. | 25,289 | 94 | Ref. | Ref. |
| Medium | 28,762 | 83 | 0.81 | 0.60 to 1.08 | 26,222 | 76 | 0.81 | 0.60 to 1.10 |
| High | 26,108 | 68 | 0.75 | 0.55 to 1.02 | 23,711 | 64 | 0.78 | 0.57 to 1.07 |
| Per increase in category | | | 0.86 | 0.74 to 1.01 |  |  | 0.88 | 0.75 to 1.03 |

CI=confidence interval; HR=hazard ratio. Hazard ratios were estimated using Cox proportional hazards regression.

^1^Adjusted for the child’s sex, parental inflammatory bowel disease, and mother’s origin, educational level, comorbidities, pre-pregnancy body mass index, and maternal antibiotic use in pregnancy (Supplementary Table 5). The analysis includes a subgroup of mother-child pairs with valid data on maternal diet and maternal antibiotic use in pregnancy (n=82,627)

^2^Adjusted for the child’s sex, parental inflammatory bowel disease, and mother’s origin, educational level, comorbidities, pre-pregnancy body mass index, and child’s antibiotic use by 18 months of age (Supplementary Table 5). The analysis includes a subgroup of mother-child pairs with valid data on maternal diet and child’s antibiotic use by 18 months of age (n=75,222).

**Supplementary Table 13.** Sensitivity analysis of maternal diet diversity, quality and intakes of specific food groups in pregnancy and the offspring’s risk of childhood-onset inflammatory bowel disease <18 years (n=263)

|  | **Unadjusted** | | | | | **Adjusted** | |
| --- | --- | --- | --- | --- | --- | --- | --- |
| **Diet exposure** | **Incidence rate**  **per 100,000 PY** | **N** | **n event** | **HR** | **95% CI** | **HR^1^** | **95% CI** |
| Diet quality | |  |  |  |  |  |  |
| Low | 21.6 (17.6 to 26.3) | 28,374 | 99 | Ref. | Ref. | Ref. | Ref. |
| Medium | 18.6 (14.8 to 23.0) | 28,373 | 84 | 0.88 | 0.66 to 1.18 | 0.90 | 0.67 to 1.21 |
| High | 18.0 (14.2 to 22.3) | 28,382 | 80 | 0.88 | 0.65 to 1.18 | 0.91 | 0.67 to 1.24 |
| Diet diversity | |  |  |  |  |  |  |
| Low | 23.2 (19.0 to 28.0) | 28,623 | 107 | Ref. | Ref. | Ref. | Ref. |
| Medium | 18.3 (14.6 to 22.5) | 29,603 | 86 | 0.80 | 0.60 to 1.07 | 0.81 | 0.60 to 1.08 |
| High | 16.5 (12.9 to 20.9) | 26,903 | 70 | 0.74 | 0.55 to 1.00 | 0.77 | 0.57 to 1.05 |
| Red meat |  |  |  |  |  |  |  |
| Low | 16.7 (13.1 to 20.9) | 28,297 | 74 | Ref. | Ref. | Ref. | Ref. |
| Medium | 21.4 (17.4 to 26.1) | 28,416 | 97 | 1.25 | 0.92 to 1.68 | 1.29 | 0.94 to 1.76 |
| High | 20 (16.1 to 24.6) | 28,416 | 92 | 1.13 | 0.84 to 1.54 | 1.14 | 0.83 to 1.57 |
| White meat |  |  |  |  |  |  |  |
| Low | 23.0 (18.8 to 27.8) | 28,298 | 106 | Ref. | Ref. | Ref. | Ref. |
| Medium | 16.4 (12.8 to 20.5) | 28,415 | 74 | 0.74 | 0.55 to 1.00 | 0.72 | 0.53 to 0.99 |
| High | 18.8 (14.9 to 23.3) | 28,416 | 83 | 0.89 | 0.67 to 1.19 | 0.89 | 0.66 to 1.20 |
| Fatty fish |  |  |  |  |  |  |  |
| Low | 20.0 (16.1 to 24.5) | 28,297 | 91 | Ref. | Ref. | Ref. | Ref. |
| Medium | 18.2 (14.5 to 22.6) | 28,417 | 82 | 0.94 | 0.69 to 1.26 | 0.97 | 0.71 to 1.33 |
| High | 20.0 (16.1 to 24.6) | 28,415 | 90 | 1.04 | 0.77 to 1.39 | 1.10 | 0.81 to 1.49 |
| Lean fish and seafood | |  |  |  |  |  |  |
| Low | 21.9 (17.8 to 26.7) | 28,297 | 98 | Ref. | Ref. | Ref. | Ref. |
| Medium | 16.2 (12.7 to 20.4) | 28,416 | 73 | 0.74 | 0.54 to 1.00 | 0.76 | 0.56 to 1.04 |
| High | 20.1 (16.2 to 24.6) | 28,416 | 92 | 0.88 | 0.66 to 1.17 | 0.91 | 0.68 to 1.22 |
| Dairy |  |  |  |  |  |  |  |
| Low | 17.3 (13.7 to 21.6) | 28,297 | 78 | Ref. | Ref. | Ref. | Ref. |
| Medium | 23.6 (19.4 to 28.6) | 28,416 | 107 | 1.36 | 1.01 to 1.82 | 1.40 | 1.04 to 1.89 |
| High | 17.2 (13.6 to 21.5) | 28,416 | 78 | 0.98 | 0.72 to 1.35 | 1.00 | 0.72 to 1.39 |
| Fruits |  |  |  |  |  |  |  |
| Low | 23.1 (18.9 to 28.0) | 28,297 | 105 | Ref. | Ref. | Ref. | Ref. |
| Medium | 15.3 (11.9 to 19.4) | 28,416 | 69 | 0.68 | 0.50 to 0.92 | 0.69 | 0.51 to 0.94 |
| High | 19.8 (15.9 to 24.3) | 28,416 | 89 | 0.88 | 0.66 to 1.17 | 0.90 | 0.67 to 1.21 |
| Vegetables |  |  |  |  |  |  |  |
| Low | 20.5 (16.5 to 25.1) | 28,297 | 93 | Ref. | Ref. | Ref. | Ref. |
| Medium | 17.0 (13.4 to 21.3) | 28,416 | 77 | 0.85 | 0.63 to 1.14 | 0.82 | 0.60 to 1.11 |
| High | 20.7 (16.7 to 25.4) | 28,416 | 93 | 1.05 | 0.78 to 1.40 | 0.98 | 0.73 to 1.33 |
| Whole grains |  |  |  |  |  |  |  |
| Low | 23.9 (19.7 to 28.9) | 28,327 | 110 | Ref. | Ref. | Ref. | Ref. |
| Medium | 13.4 (10.2 to 17.2) | 28,387 | 60 | 0.58 | 0.42 to 0.80 | 0.59 | 0.42 to 0.82 |
| High | 20.8 (16.8 to 25.4) | 28,415 | 93 | 0.91 | 0.69 to 1.21 | 0.98 | 0.73 to 1.31 |
| Refined grains |  |  |  |  |  |  |  |
| Low | 20.7 (16.7 to 25.4) | 28,297 | 92 | Ref. | Ref. | Ref. | Ref. |
| Medium | 17.9 (14.2 to 22.3) | 28,417 | 81 | 0.84 | 0.63 to 1.14 | 0.79 | 0.58 to 1.08 |
| High | 19.6 (15.7 to 24.0) | 28,415 | 90 | 0.89 | 0.66 to 1.19 | 0.88 | 0.64 to 1.19 |
| Fat-and salt-dense food | |  |  |  |  |  |  |
| Low | 19.7 (15.9 to 24.3) | 28,754 | 90 | Ref. | Ref. | Ref. | Ref. |
| Medium | 17.5 (13.9 to 21.7) | 29,126 | 81 | 0.88 | 0.65 to 1.19 | 0.80 | 0.58 to 1.09 |
| High | 21.1 (17.0 to 25.9) | 27,249 | 92 | 1.05 | 0.78 to 1.41 | 0.96 | 0.71 to 1.31 |
| Sugary foods and drinks | |  |  |  |  |  |  |
| Low | 17.3 (13.6 to 21.6) | 28,297 | 77 | Ref. | Ref. | Ref. | Ref. |
| Medium | 17.8 (14.1 to 22.1) | 28,416 | 80 | 1.02 | 0.74 to 1.39 | 0.99 | 0.72 to 1.37 |
| High | 23.0 (18.8 to 27.8) | 28,416 | 106 | 1.25 | 0.93 to 1.69 | 1.24 | 0.91 to 1.68 |

CI=confidence interval; HR=hazard ratio; PY=person-year. Incidence rates were estimated using Poisson regression.

Hazard ratios were estimated using Cox proportional hazards regression.

^1^Adjusted for the child’s sex, parental inflammatory bowel disease as well as the mother’s origin, education level, comorbidities, and pre-pregnancy body mass index (Supplementary Table 5).

# **SUPPLEMENTARY FIGURES**


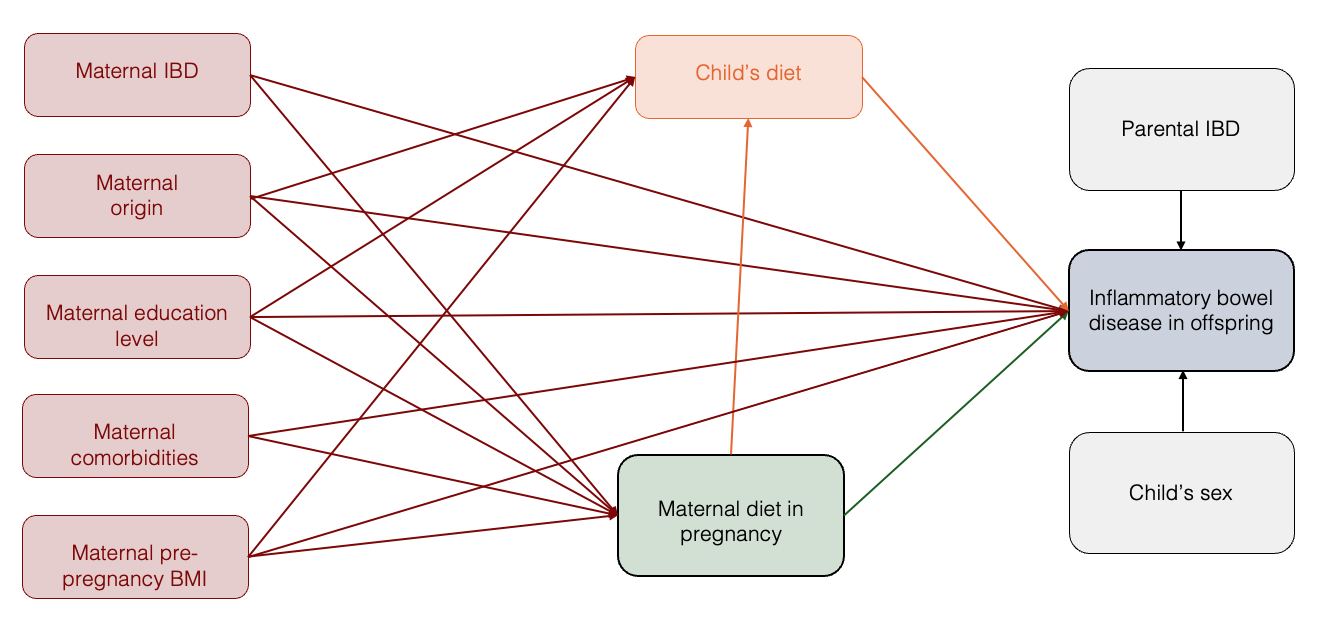


**Supplementary Figure 1.** Directed acyclic graph of potential confounders (red), mediators (orange), and ancestors (white) of IBD. Previous studies suggest an influencing effect from maternal IBD [7], origin [8], education level [9], comorbidities [10, 11], body mass index (BMI) [12], and paternal IBD [7].

# **REFERENCES**

1. Magnus P, Birke C, Vejrup K, Haugan A, Alsaker E, Daltveit AK, et al. Cohort Profile Update: The Norwegian Mother and Child Cohort Study (MoBa). Int J Epidemiol. 2016;45(2):382-8.

2. Borge TC, Brantsæter AL, Caspersen IH, Meltzer HM, Brandlistuen RE, Aase H, et al. Estimating the Strength of Associations Between Prenatal Diet Quality and Child Developmental Outcomes: Results From a Large Prospective Pregnancy Cohort Study. Am J Epidemiol. 2019;188(11):1902-12.

3. Borge TC, Biele G, Papadopoulou E, Andersen LF, Jacka F, Eggesbø M, et al. The associations between maternal and child diet quality and child ADHD - findings from a large Norwegian pregnancy cohort study. BMC Psychiatry. 2021;21(1):139.

4. Mouratidou N, Malmborg P, Järås J, Sigurdsson V, Sandström O, Fagerberg UL, et al. Identification of Childhood-Onset Inflammatory Bowel Disease in Swedish Healthcare Registers: A Validation Study. Clin Epidemiol. 2022;14:591-600.

5. Everhov Å H, Halfvarson J, Myrelid P, Sachs MC, Nordenvall C, Söderling J, et al. Incidence and Treatment of Patients Diagnosed With Inflammatory Bowel Diseases at 60 Years or Older in Sweden. Gastroenterology. 2018;154(3):518-28.e15.

6. Guo A, Ludvigsson J, Brantsæter AL, Klingberg S, Östensson M, Størdal K, et al. Early-life diet and risk of inflammatory bowel disease: a pooled study in two Scandinavian birth cohorts. Gut. 2024;73(4):590-600.

7. Santos MPC, Gomes C, Torres J. Familial and ethnic risk in inflammatory bowel disease. Ann Gastroenterol. 2018;31(1):14-23.

8. Misra R, Limdi J, Cooney R, Sakuma S, Brookes M, Fogden E, et al. Ethnic differences in inflammatory bowel disease: Results from the United Kingdom inception cohort epidemiology study. World J Gastroenterol. 2019;25(40):6145-57.

9. Sigvardsson I, Størdal K, Östensson M, Guo A, Ludvigsson J, Mårild K. Childhood Socioeconomic Characteristics and Risk of Inflammatory Bowel Disease: A Scandinavian Birth Cohort Study. Inflamm Bowel Dis. 2023.

10. Mårild K, Söderling J, Lebwohl B, Green PHR, Pinto-Sanchez MI, Halfvarson J, et al. Association of Celiac Disease and Inflammatory Bowel Disease: A Nationwide Register-Based Cohort Study. Am J Gastroenterol. 2022;117(9):1471-81.

11. Chen Y, Chen L, Xing C, Deng G, Zeng F, Xie T, et al. The risk of rheumatoid arthritis among patients with inflammatory bowel disease: a systematic review and meta-analysis. BMC Gastroenterol. 2020;20(1):192.

12. Abel MH, Caspersen IH, Sengpiel V, Jacobsson B, Meltzer HM, Magnus P, et al. Insufficient maternal iodine intake is associated with subfecundity, reduced foetal growth, and adverse pregnancy outcomes in the Norwegian Mother, Father and Child Cohort Study. BMC Med. 2020;18(1):211.
